# Physical activity, aerobic fitness and parental socio-economic position among adolescents: the German Health Interview and Examination Survey for Children and Adolescents 2003–2006 (KiGGS)

**DOI:** 10.1186/1479-5868-11-43

**Published:** 2014-03-22

**Authors:** Jonas D Finger, Gert B M Mensink, Winfried Banzer, Thomas Lampert, Thorkild Tylleskär

**Affiliations:** 1 Department of Epidemiology and Health Monitoring, Robert Koch Institute, Berlin, Germany; 2 Institute of Sports Science, Goethe University Frankfurt, Frankfurt am Main, Germany; 3 Centre for International Health, Department of Global Public Health and Primary Care, University of Bergen, Bergen, Norway

**Keywords:** Socio-economic position, Physical activity, Aerobic fitness, Adolescents, Germany

## Abstract

**Background:**

The positive association between parental socio-economic position (PSEP) and health among adolescents may be partly explained by physical activity behaviour. We investigated the associations between physical activity, aerobic fitness and PSEP in a population based sample of German adolescents.

**Methods:**

5,251 participants, aged 11–17 years, in the German Health Interview and Examination Survey for Children and Adolescents 2003–2006 (KiGGS) underwent a sub-maximal cycle ergometer test and completed a questionnaire obtaining information on physical activity and media use. The associations between physical activity, media use, aerobic fitness and PSEP were analysed with multivariate logistic regression models for boys and girls separately. Odds ratios (ORs) of PSEP (education, occupation and income) on the outcomes were calculated adjusted for age, region, and other influencing factors.

**Results:**

Parental education was more strongly associated with the outcome variables than parental occupation and income. After adjusting for age and region, a higher parental education level was associated with better aerobic fitness – with an OR of 1.5 (95% CI 1.2-1.9) for girls whose parents had secondary education and 1.9 (1.4-2.5) for girls whose parents had tertiary education compared to girls whose parents had primary education. The corresponding ORs for boys were 1.3 (1.0-1.6) and 1.6 (1.2-2.1), respectively. Higher parental education level was associated with lower media use: an OR of 2.1 (1.5-3.0) for girls whose parents had secondary education and 2.7 (1.8-4.1) for girls whose parents had primary education compared to girls whose parents had tertiary education. The corresponding ORs for boys were 1.5 (1.2-1.9) and 1.9 (1.5-2.5), respectively. Higher parental education level was associated with a higher physical activity level only among girls: an OR of 1.3 (1.0-1.6) for girls whose parents had secondary education and 1.2 (0.9-1.5) for girls whose parents had tertiary education compared to girls whose parents had primary education. The corresponding ORs for boys were 0.9 (0.8-1.2) and 0.8 (0.6-1.0), respectively.

**Conclusions:**

Adolescents of parents with low SEP showed a lower level of aerobic fitness and higher levels of media use than adolescents of parents with higher SEP. Health-promotion interventions need to reach adolescents of parents with low PSEP and stimulate physical activity.

## Introduction

High physical activity and aerobic/cardiorespiratory fitness levels in adolescence are related to better health in adolescence and early adulthood [1-3] and to an increased probability that a physically active lifestyle will be followed for the duration of the life span [4]. Studies also show that physical activity behaviour in adolescence mediates, in part, socio-economic differences in adolescent health [5], furthermore, that the consideration of socio-economic factors helps to understand differences in the physical activity socialisation of adolescents [6,7]. Although socio-economic influences are described as hardly modifiable correlates of physical activity behaviour, they play an important role in targeting and directing health promotion interventions [8]. In recent reviews the observed relationships between parental socio-economic position (PSEP) and physical activity and fitness among adolescents are inconsistent [8-12]. In fact, most studies reveal a positive association or no association. Also the level of electronic media use, as an indicator of sedentary behaviour, is observed to be inversely related to PSEP in adolescence [13]. Indicators of parental education, occupation or income are usually used to assess PSEP. Although they are related to each other, they also measure different aspects of PSEP. It would be interesting to know which of those PSEP indicators are more important than others in influencing adolescents’ physical activity behaviour. As far as we know, no study has examined the independent associations between parental education, occupation and income and physical activity and aerobic fitness among German adolescents in a population based sample. When investigating these associations it is important to also examine the role of Body Mass Index (BMI), physical wellbeing and parental support for physical activity, as studies suggest that those factors are related to PSEP [14-16] as well as being perceived barriers and/or motivations for engaging in physical activity [8,17-21].

The aim of this study is to investigate the associations of PSEP (education, occupation and income) and leisure-time physical activity, media use, aerobic fitness and total energy expenditure among adolescents in Germany. Furthermore, the aim is to examine the role of variables which may influence those associations, such as parental support for leisure time activity, physical wellbeing and BMI. The comprehensive data of the German Health Interview and Examination Survey for Children and Adolescents 2003–2006 (KiGGS) allowed us to investigate these associations.

## Methods

### Study design and participants

KiGGS is a national representative, cross-sectional survey with data collected from May 2003 until May 2006. The overall response rate of KiGGS was 66.6% [22]. The total KiGGS sample included 17,641 children and adolescents aged between 0 and 17. Using a stratified multi-stage probability sampling strategy, persons (0–17 years) were randomly selected from local population registries in 167 sample points (clusters), which were selected according to the structure of federal states and municipalities of the Federal Republic of Germany. The parents of the selected participants were also invited. The method is described in detail elsewhere [22]. The study protocol was approved by the Charité Universitätsmedizin Berlin ethics committee and the Federal Office for the Protection of Data [22].

The participants were informed about the study goals, data protection protocols and the interview and examination processes. All participants gave informed oral (11–13 years) or written (14–17 years) assent and one parent signed an informed written consent. Each participant underwent a physical examination; body weight and height was measured in a standardized way using calibrated instruments. The survey involved questionnaires filled out by the participants who were aged 11 years and older and questionnaires filled in by the parents. Participants aged 11 years and older also performed a standardized sub-maximal cycle ergometer test to assess aerobic fitness.

We included KiGGS participants 11 to 17 years old (hereafter uniformly referred to as ‘adolescents’), since this age group had the specific information on physical activity and aerobic fitness. After the exclusion of individuals with missing data (Additional file 1: Table S6) the final sample comprised 5,251 adolescents, 2,677 boys and 2,574 girls. The participation rate for the cycle ergometer test was 87% and the item response rate for each of the questionnaire items was 97%.

### Variable definitions

#### *Physical activity*

Information on *‘leisure time physical activity’* was assessed with the questions: ‘In your leisure time, how often are you physically active in such a way that you really start to sweat or get out of breath (e.g. exercising, bicycling etc.)?’ Possible answers were: ‘nearly every day’ , ‘3-5 times a week’ , ‘about 1–2 times a week’ , ‘about 1–2 times a month’ or ‘never’. The following question was: ‘About how many hours is that approximately per week? __ __’.

*‘Media use’* was assessed with the question: ‘How much time do you spend on average per day doing the following? (1) Television/video, (2) video games, (3) computer/internet, (4) listening to music, (5) using cell phone’, with answer categories: ‘not at all’ , ‘about 30 minutes’ , ‘about 1–2 hours’ , ‘about 3–4 hours’ , ‘more than 4 hours’. A media use index was calculated by cumulating the amount of time spent on a daily basis with the respective activities.

An index of *‘total energy expenditure’* in 24 hours was calculated from information on ‘leisure-time physical activity’ , ‘media use’ and ‘sleeping time’. Metabolic equivalent values (MET) were assigned to the activity categories, 0.9 MET for sleeping time, 1.3 MET for media use, 8 MET for leisure time physical activity [23]. It was assumed that the remainder of the 24 hour period was spent on average with ‘light activities’ for which a MET value of 2.5 per hour was assigned [23]. The respective activity scores were summed up into a summary score of ‘total energy expenditure’ in MET hours per 24 hours. The score is a rough estimate of energy expenditure and was used to rank individuals.

#### *Aerobic fitness*

*‘Aerobic fitness’* was measured by means of a standardized sub-maximal cycle ergometer test. The test protocol started with a workload of 0.5 watt per kg body weight and was incrementally increased every 2 minutes by another 0.5 watt per kg bodyweight [24]. The heart rate was monitored and recorded before the test and at the end of each workload stage using a computer. A 5-minute recovery period was initialized after the stage in which a heart rate of 180 heart beats per minute was exceeded. The *Physical Work Capacity* at a heart-rate threshold of 170 beats per minute (PWC170) was calculated using the mathematical approach of interpolation. PWC170 values were then divided by the body weight of the test person. The methodology of PWC170 was described in detail elsewhere [25,26]. Studies showed that the PWC170 is a valid indicator for predicting maximal oxygen uptake (VO_2_max) which is seen as the reference measure for aerobic fitness [27]; the reported correlation coefficients range between 0.66 and 0.84 [28-31].

In order to avoid questionable linearity assumptions for the relations between PSEP and physical activity and fitness outcomes, which are given in the context of linear regression analysis, all specified indices were recoded into binary variables using a standardised procedure of ranking individuals by calculating quintiles. The sample was divided into two groups, 40% vs. 60%, for boys and girls separately. The label ‘high’ was assigned to the upper 40% of the respective distributions. The following cut off points, upper limits of the 3^rd^ quintiles, were used: ‘*high aerobic fitness’ *, 2.4 watt per kg body weight for boys and 1.9 for girls; ‘*high leisure time activity’ *, 7 hours per week for boys and 4 for girls; ‘*high media use’* 6 hours per day for boys and girls; and ‘*high total energy expenditure’* within 24 hours, 46.3 MET hours for boys and 44.3 for girls.

#### *Socio-economic position*

*‘Parental education’* was obtained with two questions asking one parent about the highest school certificate and the highest vocational training certificate accomplished by the mother and father of the participant. A categorical education variable (primary, secondary, tertiary education) was generated for both parents separately by applying a revised version of the ‘Comparative Analysis of Social Mobility in Industrial Nation’ (CASMIN) classification of education for Germany [32]. If information was available for both parents, the highest education level of both parents was used to define ‘parental education’ level.

*‘Household equivalent income’* was assessed based on two questions asking about the households’ approximate monthly net income and the number of persons living permanently in the household. The household net equivalent income variable was constructed by assigning need-specific weights to the household members (OECD-modified scale: head of household = 1, additional adult household members = 0.5, children = 0.3 [33,34]), calculating the household size, and dividing the monthly net income by the household size. A categorical household income level variable was created by calculating tertiles of the ‘household-equivalent income’ variable (low, middle, high).

*‘Parental occupation’* for each parent was measured with a question asking about the ‘current or last professional position’. A categorical occupation status variable was constructed according to a revised version of the ‘Occupational Prestige in Comparative Perspective’ approach for Germany to categorising respondents into three groups of occupation status (low, middle, high) [35]. The highest occupation status of any of the parents was used to define ‘parental occupation’ status.

#### *Personal and socio-environmental variables*

*‘BMI-for-age’* was calculated for boys and girls separately using sex- and age-specific BMI reference z-scores of the World Health Organization (WHO) [36] with the following cut off points: ‘below -2 Z’ , ‘-2 to -1 Z’ , ‘-1 to +1 Z’ , ‘+1 to +2 Z’ , and ‘above +2 Z’ [37,38].

*‘Physical wellbeing’* was assessed with the questions: ‘Now I would like to know something about your body: In the last week…; …I felt sick, …I had pain, …I was tired and worn out, …I had a lot of power and endurance’; with the following answer options: ‘never’ , ‘rarely’ , ‘sometimes’ , ‘often’ , ‘always’. A sum score of the physical wellbeing items was categorised in tertiles (low, middle, high). The physical wellbeing index is a subscale of the KINDL-R instrument which measures health related quality of life in young people with acceptable validity; the reported correlation coefficient between the physical wellbeing indices of KINDL-R and the comprehensive KIDSCREEN questionnaire was 0.45 [39,40].

*‘Parental support for leisure time activity’* was assessed with the question: ‘How is it in your family? In the evening and at weekends we rather stay at home than doing leisure activities together’. With answer possibilities: ‘disagree’; ‘somewhat disagree’; ‘somewhat agree’; ‘agree’. The question is a sub-item of the ‘Familienklimaskalen’ (FKS) [41], which is a translated and slightly adapted German version of the family environment scales (FES) [42].

### Statistical analysis

The statistical analysis was performed with STATA SE 12.0. The cluster structure of the multi-stage sample was accounted for by using survey design procedures. These procedures lead to wider confidence intervals compared to standard statistical procedures, which assume simple random sampling. Possible influencing factors for the associations investigated were initially selected based on knowledge and theories from literature, and inference statistics were then used to clarify their statistical significance. Confounding and interaction on the associations between parental education level and adolescents’ physical activity and fitness outcomes were tested by fitting stepwise logistic regression models (*Model 1*: outcome and exposure variable, *Model 2*: Model 1 + covariate, *Model 3*: Model 2 + interaction term of exposure*covariate). Estimations of each model were stored at each stage and tested for model fit using a likelihood-ratio test (lrtest) by comparing the post-estimations of the respective models. If the lrtest was significant (95% level of confidence) comparing Model 2 and 3, the covariate was considered to be an effect modifier and sub-group analyses were performed. If the lrtest was significant comparing Model 1 and 2 and the covariate was associated with the exposure, the covariate was considered to be a potential confounder. The age- and region-adjusted associations between PSEP and physical activity and fitness variables in the basic models were subsequently adjusted for potential confounders for the respective associations. When adjusting for covariates, we used the following age group strata: 11, 12, 13, 14, 15, 16, 17 years; the region strata: ‘former East Germany’ and ‘former West Germany’ , the ‘BMI-for-age’ strata, ‘below -2 Z’ , ‘-2 to -1 Z’ , ‘-1 to +1 Z’ , ‘+1 to +2 Z’ , ‘above +2 Z’; the strata of ‘low parental support for leisure time activity’ ‘disagree’ , ‘somewhat disagree’ , ‘somewhat agree’ , ‘agree’; as well as quintiles of the ‘physical wellbeing’ score. Missing values of the covariates (e.g. parental occupation, household income) were integrated into the statistical analyses by constructing a separate category for missing values (the numbers are shown in Table 1).

**Table 1:** Description of participants and means of the outcome indicators according to selected key variables, boys and girls 11–17 years

	**Study sample**		**Leisure time activity**	**Media use**	**PWC170**^**a**^	**Total energy expenditure**^**b**^
**Characteristics**	**n**	**%**	**Mean hours/week**	**Mean hours/day**	**Mean watt/kg body weight**	**Mean MET/24 hours**
**Total**	5251		6.3	5.9	2.1	44.2
**Age (years)**						
11 - 13	2401	46	6.5	4.8	2.0	45.7
14 - 15	1522	29	6.4	6.7	2.1	43.4
16 - 17	1328	25	5.6	7.0	2.1	42.5
**Sex**						
boys	2677	51	7.9	5.9	2.3	45.5
girls	2574	49	4.6	5.9	1.9	42.9
**Region in Germany**						
Former East	1756	34	6.2	6.3	2.1	43.7
Former West	3495	66	6.3	5.7	2.1	44.5
**Parental education**						
Primary	972	19	6.7	6.5	2.0	43.8
Secondary	2835	54	6.4	6.1	2.1	44.1
Tertiary	1444	28	5.7	5.1	2.1	44.8
**Parental occupation**						
Low	1526	29	6.9	6.8	2.0	43.6
Middle	1708	33	6.1	5.7	2.1	44.4
High	1871	36	5.9	5.3	2.1	44.6
Missing	146	3	6.4	6.9	2.0	43.2
**Household income**						
Low	1680	32	6.5	6.3	2.0	44.0
Middle	1452	26	6.3	5.9	2.1	44.3
High	1693	32	5.9	5.5	2.1	44.5
Missing	426	8	6.6	6.2	2.1	44.1
**BMI-for-age (WHO z-scores)**						
Below -2 Z	83	2	5.4	5.4	2.3	44.2
-2 to -1 Z	478	9	6.5	5.3	2.2	45.1
-1 to +1 Z	3310	63	6.1	5.9	2.1	44.2
+1 to +2 Z	961	18	6.7	6.1	1.9	44.4
Above +2 Z	419	8	6.5	6.6	1.6	43.5
**Physical wellbeing**						
Low	1902	36	5.7	6.3	2.0	43.3
Middle	1520	29	6.2	5.8	2.1	44.3
High	1689	32	7.1	5.5	2.1	45.3

^a^PWC170, physical work capacity at a heart rate of 170 heart beats per minute. ^b^Energy expenditure assessed on the basis of self-reported activities within 24 h, expressed in metabolic equivalents (MET) kcal/kg, 1 MET = a person’s caloric consumption at complete rest.

## Results

### Participants

According to the response analysis (see Additional file 1: Table S5) parents of the respondents reported, on average, higher levels of education compared to parents of the non-respondents. Respondents were on average younger and had a lower BMI and health state compared to the non-respondents. Respondents with missing data for the key variables (parental education, physical activity or fitness variables) were therefore excluded from the presented analyses and showed a lower level of physical wellbeing, a higher BMI and media use and their parents had lower education compared to respondents with complete information (see Additional file 1: Table S6).

In the final study sample, 19% of the parents had a low level of education and 28% had a high level of education. Adolescents in higher age groups showed statistically significant (95% level of confidence) lower levels of both ‘leisure time physical activity’ and ‘total energy expenditure’ and higher levels of ‘media use’ than their peers in lower age groups. Moreover, boys showed higher levels of leisure time activity, total energy expenditure and aerobic fitness than girls (Table 1).

### Multivariate analyses

The age and region adjusted analysis revealed that adolescents of parents with higher levels of education, occupation and income were more likely to show high levels of aerobic fitness and less likely to report high levels of media use compared to their peers with parents of lower socio-economic status (Tables 2, 3). Amongst girls positive associations between PSEP and leisure time physical activity and total energy expenditure were also observed in the basic models.

**Table 2:** Stepwise adjusted odds ratios (OR) of physical activity and media use according to parental education, boys and girls 11–17 years

	**High leisure time activity**		**High media use**	
	**Basic model**^**a**^	**Final model**^**b**^	**Basic model**^**a**^	**Final model**^**c**^
	**OR 95% CI**	**OR 95% CI**	**OR 95% CI**	**OR 95% CI**
**Boys (n = 2677)**				
**Parental education**				
Primary	1.0	1.0	1.0	1.0
Secondary	0.9 (0.8-1.2)	0.9 (0.7-1.2)	0.8 (0.6-1.0)	0.9 (0.7-1.2)
Tertiary	0.8 (0.6-1.0)	0.8 (0.6-1.1)	0.5 (0.4-0.7)	0.7 (0.5-0.9)
**Parental occupation**				
Low	1.0	1.0	1.0	1.0
Middle	0.8 (0.7-1.0)	0.8 (0.7-1.0)	0.5 (0.4-0.7)	0.6 (0.5-0.7)
High	0.8 (0.6-1.0)	0.9 (0.7-1.1)	0.5 (0.4-0.6)	0.6 (0.5-0.8)
**Household income**				
Low	1.0	1.0	1.0	1.0
Middle	0.9 (0.8-1.1)	1.0 (0.8-1.2)	0.8 (0.6-1.0)	0.9 (0.7-1.2)
High	0.9 (0.7-1.1)	0.9 (0.7-1.2)	0.7 (0.5-0.8)	1.0 (0.8-1.3)
**Girls (n = 2574)**				
**Parental education**				
Primary	1.0	1.0	1.0	1.0
Secondary	1.3 (1.0-1.6)	1.2 (0.9-1.5)	0.7 (0.6-0.9)	0.8 (0.6-1.1)
Tertiary	1.2 (0.9-1.5)	1.0 (0.7-1.3)	0.3 (0.3-0.5)	0.5 (0.3-0.6)
**Parental occupation**				
Low	1.0	1.0	1.0	1.0
Middle	1.1 (0.9-1.4)	1.1 (0.9-1.4)	0.5 (0.4-0.7)	0.7 (0.6-0.9)
High	1.3 (1.0-1.6)	1.2 (0.9-1.6)	0.5 (0.4-0.6)	0.7 (0.6-1.0)
**Household income**				
Low	1.0	1.0	1.0	1.0
Middle	0.9 (0.7-1.1)	0.9 (0.7-1.1)	0.7 (0.6-0.9)	0.9 (0.7-1.2)
High	1.2 (1.0-1.5)	1.1 (0.9-1.4)	0.6 (0.5-0.7)	1.0 (0.8-1.3)

^a^Model adjusted for age groups and region strata East–west Germany (separate models for education, occupation and income). ^b^Adjusted as the basic model and also for ‘physical wellbeing’ , ‘parental support for leisure time activity’ among boys, and ‘BMI-for-age’ among boys (education, occupation and income in combined model). ^c^Adjusted as the basic model and also for ‘BMI-for-age’ among girls, ‘physical wellbeing’ among girls and ‘parental support for leisure time activity’ (education, occupation and income in combined model).

**Table 3:** Stepwise adjusted odds ratios (OR) of physical work capacity and total energy expenditure according to parental education, boys and girls 11–17 years

	**High aerobic fitness**		**High total energy expenditure**	
	**Basic model**^**a**^	**Final model**^**b**^	**Basic model**^**a**^	**Final model**^**c**^
	**OR 95% CI**	**OR 95% CI**	**OR 95% CI**	**OR 95% CI**
**Boys (n = 2677)**				
**Parental education**				
Primary	1.0	1.0	1.0	1.0
Secondary	1.3 (1.0-1.6)	1.1 (0.8-1.4)	1.1 (0.8-1.3)	1.0 (0.8-1.2)
Tertiary	1.6 (1.2-2.1)	1.3 (1.0-1.8)	1.2 (0.9-1.5)	1.1 (0.8-1.5)
**Parental occupation**				
Low	1.0	1.0	1.0	1.0
Middle	1.4 (1.2-1.7)	1.3 (1.0-1.5)	1.2 (1.0-1.5)	1.2 (1.0-1.5)
High	1.3 (1.1-1.6)	1.1 (0.8-1.4)	1.1 (0.9-1.4)	1.1 (0.9-1.4)
**Household income**				
Low	1.0	1.0	1.0	1.0
Middle	1.2 (1.0-1.5)	1.0 (0.8-1.3)	1.1 (0.9-1.4)	1.0 (0.8-1.3)
High	1.2 (1.0-1.5)	1.0 (0.8-1.3)	1.1 (0.9-1.3)	0.9 (0.7-1.2)
**Girls (n = 2574)**				
**Parental education**				
Primary	1.0	1.0	1.0	1.0
Secondary	1.5 (1.2-1.9)	1.4 (1.1-1.8)	1.5 (1.2-1.9)	1.3 (1.1-1.7)
Tertiary	1.9 (1.4-2.5)	1.4 (1.0-1.9)	2.4 (1.8-3.1)	1.8 (1.4-2.4)
**Parental occupation**				
Low	1.0	1.0	1.0	1.0
Middle	1.3 (1.1-1.6)	1.1 (0.8-1.3)	1.4 (1.1-1.7)	1.2 (0.9-1.5)
High	1.7 (1.3-2.0)	1.2 (1.0-1.6)	1.8 (1.4-2.3)	1.3 (1.0-1.7)
**Household income**				
Low	1.0	1.0	1.0	1.0
Middle	1.2 (1.0-1.5)	1.0 (0.8-1.3)	1.2 (1.0-1.5)	1.0 (0.8-1.3)
High	1.5 (1.2-1.8)	1.1 (0.8-1.4)	1.6 (1.2-2.0)	1.0 (0.8-1.3)

^a^Model adjusted for age groups and region strata East–west Germany (separate models for education, occupation and income). ^b^Adjusted as the basic model and also for ‘BMI-for-age’, ‘physical wellbeing’ and ‘parental support for leisure time activity’ among girls (education, occupation and income in combined model). ^c^Adjusted as the basic model and also for ‘BMI-for-age’ among boys, ‘physical wellbeing’ and ‘parental support for leisure time activity’ (education, occupation and income in combined model).

#### *Leisure time physical activity*

‘Physical wellbeing’, ‘parental support for leisure time activity’ (only among boys) and ‘BMI-for-age’ (only among boys) were considered to be potential confounders of the association between ‘parental education’ and ‘leisure time physical activity’ (Table 2). No significant association between PSEP and ‘leisure time activity’ remained after adjustment.

#### *Media use*

‘Physical wellbeing’ (only among girls), ‘parental support for leisure time activity’ and ‘BMI-for-age’ (only among girls) were considered to be potential confounders of the association between ‘parental education’ and ‘media use’ (Table 2). After multivariate adjustment a significant negative association remained between ‘parental education’ and ‘media use’ and ‘parental occupation’ and ‘media use’ among both boys and girls.

#### *Aerobic fitness*

‘BMI-for-age’ , ‘physical wellbeing’ and ‘parental support for leisure time activity’ (only among girls) were considered to be potential confounders of the association between ‘parental education’ and ‘high aerobic fitness’ (Table 3). After adjustment, a significant positive association between ‘parental education’ and ‘aerobic fitness’ remained among girls and between ‘parental occupation’ and ‘aerobic fitness’ among boys.

#### *Total energy expenditure*

‘BMI-for-age’ (only among boys), ‘physical wellbeing’ and ‘parental support for leisure time activity’ were considered to be potential confounders of the association between ‘parental education’ and ‘total energy expenditure’ (Table 3). After adjustment a significant positive association remained between ‘parental education’ and ‘total energy expenditure’ and between ‘parental occupation’ and ‘total energy expenditure’ among girls.

#### *Subgroup analyses*

‘Leisure time physical activity’ was a significant effect modifier (95% level of confidence) for the association between ‘parental education’ and ‘aerobic fitness’ among boys (interaction term p-value: < 0.01). The association was weaker in the stratum ‘physical activity < 5 hours per week’ than in the stratum ‘physical activity ≥ 5 hours per week’.

## Discussion

As far as we know, this is the first study of independent associations of parental education, occupation and income and physical activity and aerobic fitness outcomes among adolescents in Germany. In addition, it was performed in a nationally representative sample. Girls of parents with high PSEP were more physically active in their leisure time, spent less time using electronic media, showed better aerobic fitness and had higher total energy expenditure compared to girls of parents with low PSEP. Boys of parents with high PSEP also spent less time using media and showed better aerobic fitness than boys of parents with low PSEP; however, no substantial differences were observed for leisure time physical activity and total energy expenditure. Media use was the outcome indicator which showed the strongest associations with PSEP among boys and girls. Although boys of parents with low PSEP reported slightly higher levels of leisure time physical activity, they also reported much higher durations of electronic media use and as a result they showed slightly lower levels of total energy expenditure compared to boys of parents with high PSEP.

In line with our observations, in the Health Behaviour in School-aged Children (HBSC) survey it was observed that in most of the 32 participating countries girls of parents with high socio-economic position indicated higher levels of leisure time physical activity and that this association was less clear among boys [43]. In addition, other studies observed that media use/television viewing was inversely related to PSEP [13,44], which is also in line with our observations.

Apart from individual factors (psychological and biological dispositions) etiological models also identify interpersonal factors (parental support, cultural norms and practices) and the built environment (neighborhood walkability, pedestrian safety, and access to parks, recreation and sports facilities) [45,46] as determinants of physical activity behavior in early life episodes [47].

In line with other studies [14-16], we observed that ‘BMI-for-age’ , ‘physical wellbeing’ and ‘parental support for leisure time activity’ were associated with both the PSEP and the adolescents’ physical activity and fitness variables. Furthermore, we observed that when adjusting for these factors, the effect sizes of the observed associations were reduced. Thus, these factors could be hypothesised as being mediators for the investigated relations, as they meet the criteria of transmitting the effect of the independent variable on the dependent variable and being in the causal sequence of the two-variable relation [48]. Studies suggest that concerns about body shape and weight management are the main motivations to participate in physical activity among young girls [17]. In addition, the perception of being overweight may be a barrier to becoming physically active among overweight individuals [19,49], as they often feel more discomfort when being physically active [20]. Also low physical wellbeing (e.g. feeling tired/sick, having a disease) and lack of parental support for physical activity have been identified as barriers to physical activity [17-21]. It was also shown that the parental physical activity level strongly correlates to the physical activity level of their children [8,50], thus it could be the case that the existing SEP differences in adults’ physical activity are transferred to their children. Parents who are mainly sedentary at work and therefore exercise more in leisure time (mainly highly educated) [51], perhaps stimulate their children to exercise together with them more strongly, compared to parents who do physically demanding work and may therefore be less active in leisure time (mainly low educated) [51], as they more frequently recover from physical work by ‘staying home in the evenings and the weekends’ using media for entertainment. Overall, we observed that ‘parental education’ was more strongly associated with physical activity and aerobic fitness outcomes among adolescents as compared to ‘parental occupation’ and ‘household income’. In particular ‘household income’ showed no independent effects on the investigated outcomes. These observations correspond to the findings of a study among German adults which uses the same SEP measures and also suggest that education, followed by occupation, is most strongly associated with physical activity patterns and that income plays no important role [51]. Assuming that the leisure time physical activity behaviour of children relates to that of their parents [50], it is plausible to conclude that the associations between parental education, occupation and income and physical activity and fitness among adolescents follow similar patterns as can be observed among adults.

### Limitations

The cross‒sectional study design does not allow for drawing causal inference upon the findings of this study. Furthermore, validation studies conducted among adolescents 12–17 years old have shown that physical activity questionnaires may overestimate physical activity level compared to objectively measured information using accelerometers. The validity of questionnaires seems to be lower among younger adolescents (12–14 years) compared to older adolescents (15–17 years) [52]. Physical activity level might be over reported due to social desirability [53], inaccuracies may also occur from cognitive problems in recalling physical activity behaviour or in misunderstanding of the underlying concepts of the questions. We therefore decided to use the self-reported information only to rank individuals by calculating quintiles. We performed sensitivity analysis in order to see whether the choice of cut off points may have influenced the results. When using continuous variables (linear regression) or ordinal (quintile) variables (ordered logistic regression) as the dependent variables in the models, the directions of the associations were widely the same as observed for the binary outcome variables. The exception was that the observed positive association between parental education and leisure time physical activity among girls was only borderline significant when using the continuous variable (p-value: 0.055) and the ordinal variable (p-value: 0.057). We showed a clear positive association between measured aerobic fitness and parental education among boys, however, not so for self-reported leisure time physical activity and total energy expenditure. We cannot totally exclude the possibility that the degree of over-reporting of physical activity level differed according to parental education level, which would result in some degree of misclassification bias. Therefore, we propose using more objective methods for measuring physical activity in future studies, for instance through use of accelerometers. If physical activity is assessed with questionnaires however, we suggest using domain-specific physical activity questionnaires in order to be able to reveal physical activity disparities by PSEP in specific health promotion relevant settings. Studies have shown that adolescents of parents with high PSEP more often engage in sports activities in sports clubs, whereas adolescents of low PSEP are more often physically active travelling from place to place [54]. Estimating the aerobic fitness via a sub-maximal exercise test based on heart rate parameters (PWC170) produces less accurate results than measuring the VO_2_max as the reference standard for cardiorespiratory fitness via a maximal exercise test [29-31]. However, maximal exercise tests are more expensive, because they require more safety equipment and better trained personal [55].

The relatively large group of persons with missing data for the aerobic fitness variable may however lead to a lower validity of the results. Finally, the generalisability of the results could be further compromised, since the respondents differed according to selected variables from the non-respondents.

## Conclusions

Compared to their peers with parents with high SEP, boys and girls of parents with low SEP showed lower levels of aerobic fitness and higher levels of media use. Girls of parents with low SEP also showed lower levels of leisure time physical activity and total energy expenditure. Parental education and occupation were more strongly related to adolescents’ physical activity and fitness outcomes than family income. A high BMI, low physical wellbeing and low parental support for leisure time activity seem to be barriers to engaging in physical activity, and adolescents of parents with low SEP seem to be more strongly affected by such barriers. In order to reduce health inequalities, health promotion interventions need to reach adolescents of parents with low SEP and to stimulate and remove barriers to physical activity. Parental involvement can be a crucial factor for the success of such interventions. In future studies more objective measures of physical activity and domain-specific physical activity questionnaires should be used.

## Competing interests

The authors declare that they have no competing interests.

## Authors’ contributions

JF structured and analysed the data, and wrote the first draft and the final version of the manuscript. GM and TL were involved in the design and conduction of KiGGS and contributed to the construction of several variables. WB contributed to constructing the physical activity and aerobic fitness variables. TT contributed to structuring the statistical analyses. All authors contributed to writing and revising the manuscript, and read and approved the final manuscript.

## Supplementary Material

Additional file 1 **Contains the results of the response analysis (Table S5) and the differences between the group of excluded cases and the final study sample (Table S6) as regards selected variables.** Adobe Acrobat Reader is required to access this file. Click here for file

## References

[B1] Ortega F Ruiz J Castillo M Sjöström M Physical fitness in childhood and adolescence: a powerful marker of health Int J Obes 2007 32 1 11 10.1038/sj.ijo.0803774 18043605

[B2] Hallal PC Victora CG Azevedo MR Wells JCK Adolescent physical activity and health: a systematic review Sports Med 2006 36 1019 1030 10.2165/00007256-200636120-00003 17123326

[B3] Tittlbach SA Sygusch R Brehm W Woll A Lampert T Abele AE Bös K Association between physical activity and health in German adolescents Eur J Sport Sci 2011 11 283 291 10.1080/17461391.2010.509891

[B4] Telama R Tracking of physical activity from childhood to adulthood: a review Obesity Facts 2009 2 187 195 10.1159/000222244 20054224 PMC6516203

[B5] Richter M Erhart M Vereecken CA Zambon A Boyce W Gabhainn SN The role of behavioural factors in explaining socioeconomic differences in adolescent health: a multilevel study in 33 countries Soc Sci Med 2009 69 396 403 10.1016/j.socscimed.2009.05.023 19540029

[B6] Kohl HW Hobbs KE Development of physical activity behaviors among children and adolescents Pediatrics 1998 101 549 12224661

[B7] Bourdieu P Sport and social class Soc Sci Inf 1978 17 819 10.1177/053901847801700603

[B8] Gustafson SL Rhodes RE Parental correlates of physical activity in children and early adolescents Sports Med 2006 36 79 97 10.2165/00007256-200636010-00006 16445312

[B9] Van der Horst K PAW MJ Twisk JWR Van Mechelen W A brief review on correlates of physical activity and sedentariness in youth Med Sci Sports Exerc 2007 39 1241 10.1249/mss.0b013e318059bf35 17762356

[B10] Stalsberg R Pedersen AV Effects of socioeconomic status on the physical activity in adolescents: a systematic review of the evidence Scand J Med Sci Sports 2010 20 368 383 10.1111/j.1600-0838.2009.01047.x 20136763

[B11] Batty GD Leon DA Socio-economic position and coronary heart disease risk factors in children and young people Evidence from UK epidemiological studies Eur J Pub Health 2002 12 263 272 10.1093/eurpub/12.4.263 12506501

[B12] Hanson MD Chen E Socioeconomic status and health behaviors in adolescence: a review of the literature J Behav Med 2007 30 263 285 10.1007/s10865-007-9098-3 17514418

[B13] Wijtzes AI Jansen W Kamphuis CB Jaddoe VW Moll HA Tiemeier H Verhulst FC Hofman A Mackenbach JP Raat H Increased risk of exceeding entertainment-media guidelines in preschool children from low socioeconomic background: The Generation R Study Prev Med 2012 55 325 329 10.1016/j.ypmed.2012.07.023 22890021

[B14] McMurray RG Harrell JS Deng S Bradley CB Cox LM Bangdiwala SI The influence of physical activity, socioeconomic status, and ethnicity on the weight status of adolescents Obesity 2000 8 130 139 10.1038/oby.2000.14 10757199

[B15] Sallis JF Zakarian JM Hovell MF Hofstetter CR Ethnic, socioeconomic, and sex differences in physical activity among adolescents J Clin Epidemiol 1996 49 125 134 10.1016/0895-4356(95)00514-5 8606313

[B16] Allison KR Dwyer JJM Makin S Perceived barriers to physical activity among high school students Prev Med 1999 28 608 615 10.1006/pmed.1999.0489 10404559

[B17] Allender S Cowburn G Foster C Understanding participation in sport and physical activity among children and adults: a review of qualitative studies Health Educ Res 2006 21 826 835 10.1093/her/cyl063 16857780

[B18] Daskapan A Tuzun E Eker L Perceived barriers to physical activity in university students J Sports Sci Med 2006 5 615 620 24357957 PMC3861763

[B19] Ibrahim S Karim N Oon NL Ngah WZW Perceived physical activity barriers related to body weight status and sociodemographic factors among Malaysian men in Klang Valley BMC Public Health 2013 13 275 10.1186/1471-2458-13-275 23530696 PMC3617120

[B20] Ishii K Inoue S Ohya Y Odagiri Y Takamiya T Suijo K Owen N Shimomitsu T Sociodemographic variation in the perception of barriers to exercise among Japanese adults J Epidemiol 2009 19 161 168 10.2188/jea.JE20080094 19542687 PMC3924104

[B21] Reichert F Barros A Domingues M Hallal P The role of perceived personal barriers to engagement in leisure-time physical activity Am J Public Health 2007 97 515 519 10.2105/AJPH.2005.070144 17267731 PMC1805028

[B22] Kurth BM Kamtsiuris P Hölling H Schlaud M Dölle R Ellert U Kahl H Knopf H Lange M Mensink GBM The challenge of comprehensively mapping children's health in a nation-wide health survey: design of the German KiGGS-Study BMC Public Health 2008 8 196 10.1186/1471-2458-8-196 18533019 PMC2442072

[B23] Ridley K Ainsworth B Olds T Development of a compendium of energy expenditures for youth Int J Behav Nutr Phys Act 2008 5 45 10.1186/1479-5868-5-45 18782458 PMC2564974

[B24] Rost R Lehrbuch der Sportmedizin [Textbook of Sports Medicine] 2001 Deutscher Ärzte-Verlag: Köln

[B25] Wahlund H Determinants of the physical work capacity. A physiological and clinical study with special reference to standardization of cardio-pulmonary functional test Acta Medica Scandinavica 1948 215 Suppl 83 98

[B26] Bengtsson E The work capacity in normal children, evaluated by submaximal excerise on the bicycle ergometer and compared with adults Acta Medica Scandinavica 1956 154 91 109 13313072 10.1111/j.0954-6820.1956.tb14303.x

[B27] Armstrong N Tomkinson G Ekelund U Aerobic fitness and its relationship to sport, exercise training and habitual physical activity during youth Br J Sports Med 2011 45 849 858 10.1136/bjsports-2011-090200 21836169

[B28] Boreham C Paliczka V Nichols A A comparison of the PWC170 and 20-MST tests of aerobic fitness in adolescent schoolchildren J Sports Med and Phys Fitness 1990 30 19 2366530

[B29] McMurray RG Guion WK Ainsworth BE Harrell JS Predicting aerobic power in children. A comparison of two methods J Sports Med Phys Fitness 1998 38 227 233 9830830

[B30] Rowland TW Rambusch JM Staab JS Unnithan VB Siconolfi SF Accuracy of physical working capacity (PWC170) in estimating aerobic fitness in children J Sports Med Phys Fitness 1993 33 184 188 8412055

[B31] Heyman E Briard D Dekerdanet M Gratas-Delamarche A Delamarche P Accuracy of physical working capacity 170 to estimate aerobic fitness in prepubertal diabetic boys and in 2 insulin dose conditions J Sports Med Phys Fitness 2006 46 315 321 16823364

[B32] Schroedter JH Lechert Y Lüttinger P Die Umsetzung der Bildungsklassifikation CASMIN für die Volkszählung 1970, die Mikrozensus-Zusatzerhebung 1971 und die Mikrozensen 1976–2004 [Transformation of the CASMIN education classification for the census 1970, the micro-census supplement 1971 and the micro-censuses 1976–2004] ZUMA Methodenbericht 2006 12 1 58

[B33] OECD OECD: Project on Income Distribution and Poverty. What are equivalent scales? http://www.oecd.org/eco/growth/OECD-Note-EquivalenceScales.pdf (accessed: 3 December 2011)

[B34] Hagenaars AJM De Vos K Zaidi MA Poverty statistics in the late 1980s: Research based on micro-data 1996 Luxembourg: Office for Official Publications of the European Communities

[B35] Hoffmeyer-Zlotnik J Geis A Berufsklassifikation und Messung des beruflichen Status/Prestige [Occupational classification and measurement of occupational status/prestige] ZUMA Nachrichten 2003 52 125 138

[B36] Cochrane SH Leslie J O'Hara DJ Parental education and child health: intracountry evidence Health Pol Educ 1982 2 213 250 10.1016/0165-2281(82)90011-X 10298649

[B37] Cole TJ Flegal KM Nicholls D Jackson AA Body mass index cut offs to define thinness in children and adolescents: international survey BMJ 2007 335 194 10.1136/bmj.39238.399444.55 17591624 PMC1934447

[B38] Onis M Onyango AW Borghi E Siyam A Nishida C Siekmann J Development of a WHO growth reference for school-aged children and adolescents Bull World Health Organ 2007 85 660 667 10.2471/BLT.07.043497 18026621 PMC2636412

[B39] Bullinger M Brütt D-PAL Erhart M Ravens-Sieberer U Psychometric properties of the KINDL-R questionnaire: results of the BELLA study Eur Child Adolesc Psychiatr 2008 17 125 132 10.1007/s00787-008-1014-z 19132312

[B40] Ravens-Sieberer U Bullinger M Schuhmacher J, Klaiberg A, Brähler E Der Kindl-R Fragebogen zur Erfassung der gesundheitsbezogenen Lebensqualität bei Kindern und Jugendlichen-Revidierte Form Diagnostische Verfahren zu Lebensqualität und Wohlbefinden 2003 Göttingen: Hogrefe 184 188

[B41] Schneewind K Cierpka M Die Familienklimaskalen (FKS) Familiendiagnostik 1988 Heidelberg: Springer 232 255

[B42] Moos RH Moos BS Family Environment Scale Manual 1994 Palo Alto, CA: Consulting Psychologists Press

[B43] Borraccino A Lemma P Iannotti R Zambon A Dalmasso P Lazzeri G Giacchi M Cavallo F Socio-economic effects on meeting PA guidelines: comparisons among 32 countries Med Sci Sports Exerc 2009 41 749 10.1249/MSS.0b013e3181917722 19276860 PMC2663903

[B44] Gorely T Marshall SJ Biddle SJH Couch kids: correlates of television viewing among youth Int J Behav Med 2004 11 152 163 10.1207/s15327558ijbm1103_4 15496343

[B45] Sandercock G Angus C Barton J Physical activity levels of children living in different built environments Prev Med 2010 50 193 198 10.1016/j.ypmed.2010.01.005 20083131

[B46] Lawman HG Wilson DK A review of family and environmental correlates of health behaviors in high-risk youth Obesity 2012 20 1142 1157 10.1038/oby.2011.376 22282044 PMC3360830

[B47] Bauman AE Reis RS Sallis JF Wells JC Loos RJ Martin BW Lancet Physical Activity Series Working G Correlates of physical activity: why are some people physically active and others not? Lancet 2012 380 258 271 10.1016/S0140-6736(12)60735-1 22818938

[B48] MacKinnon DP Fairchild AJ Fritz MS Mediation analysis Annu Rev Psychol 2007 58 593 10.1146/annurev.psych.58.110405.085542 16968208 PMC2819368

[B49] Ball K Crawford D Owen N Too fat to exercise? Obesity as a barrier to physical activity Aust N Z J Public Health 2000 24 331 333 10.1111/j.1467-842X.2000.tb01579.x 10937415

[B50] Aarnio M Winter T Kujala U Kaprio J Familial aggregation of leisure-time physical activity-a three generation study Int J Sports Med 1997 18 549 556 10.1055/s-2007-972680 9414080

[B51] Finger JD Tylleskär T Lampert T Mensink GBM Physical activity patterns and socioeconomic position: the German National Health Interview and Examination Survey 1998 (GNHIES98) BMC Publ Health 2012 12 1079 10.1186/1471-2458-12-1079 PMC3561273 23241280

[B52] Hagströmer M Bergman P De Bourdeaudhuij I Ortega FB Ruiz JR Manios Y Rey-Lopez J Phillipp K Von Berlepsch J Sjöström M Concurrent validity of a modified version of the International Physical Activity Questionnaire (IPAQ-A) in European adolescents: the HELENA Study Int J Obes 2008 32 S42 S48 10.1038/ijo.2008.182 19011653

[B53] Jago R Baranowski T Baranowski JC Cullen KW Thompson DI Social desirability is associated with some physical activity, psychosocial variables and sedentary behavior but not self-reported physical activity among adolescent males Health Educ Res 2007 22 438 449 16987942 10.1093/her/cyl107

[B54] Bös K Worth A Opper E Oberger J Woll A Motorik-Modul: Eine Studie zur motorischen Leistungsfähigkeit und körperlich-sportlicher Aktivität von Kindern und Jugendlichen in Deutschland [Motoric Module: A Study on the Physical Fitness and Physical Activity among Children and Adolescents in Germany] 2009 Nomos Verlag: Baden-Baden

[B55] American College of Sports Medicine ACSM's Guidelines for Exercise Testing and Prescription 2006 seventh Philadelphia: Lippincott Williams & Wilkins

