# Supplementary material for: Physical activity, aerobic fitness and parental socio-economic position among adolescents: the German Health Interview and Examination Survey for Children and Adolescents 2003–2006 (KiGGS)
Source: Int J Behav Nutr Phys Act. 2014 Mar 22;11:43. doi: 10.1186/1479-5868-11-43 (PMC3997963; doi:10.1186/1479-5868-11-43)
Supplement: Additional file 1 — Contains the results of the response analysis (Table S5) and the differences between the group of excluded cases and the final study sample (Table S6) as regards selected variables. Adobe Acrobat Reader is required to access this file. [file 1479-5868-11-43-S1.pdf]

Additional File

Table S5. Prevalence percentages/means, 95 % confidence intervals and p-values of test of proportion/t-test for selected characteristics of respondents versus non-respondents, adolescents aged 11-17, KiGGS 2003-06

	Responders (n=6813)	Non-responders (n=3142)	Test of proportion/t-test
**Mean age (years)**	13.9 (13.8-13.9)	14.3 (14.2-14.3)	P<0.001
**Sex**			
boys (%)	51.3 (50.1-52.4)	52.5 (50.7-54.2)	P=0.27
**School education of mother (%)**			
basic level	25.0 (23.9-26.0)	37.5 (35.7-39.2)	P<0.001
secondary level	47.0 (45.8-48.2)	40.2 (38.5-42.0)	P<0.001
advanced level	24.2 (23.2-25.3)	16.8 (15.5-18.1)	P<0.001
other	3.8 (3.3-4.2)	5.5 (4.7-6.4)	P<0.001
number of missing cases	208	128	
**School education of father (%)**			
basic level	31.9 (30.8-33.1)	39.2 (37.4-41.1)	P<0.001
secondary level	36.9 (35.7-38.1)	33.2 (31.4-34.9)	P<0.001
advanced level	27.3 (26.2-28.4)	21.8 (20.2-23.3)	P<0.001
other	3.9 (3.4-4.4)	5.8 (5.0-6.7)	P<0.001
number of missing cases	514	347	
**Health state of adolescent**			
'very good' health (%)	32.6 (31.5-33.7)	37.9 (36.2-39.5)	P<0.001
mean number of consultations of a health professional in the past 12 month	3.8 (3.6-3.9)	2.8 (2.6-3.0)	P<0.001
**Body Mass Index [a] (mean)**	20.2 (20.1-20.3)	20.6 (20.5-20.8)	P<0.001

[a] Body Mass Index = weight (kg) / height (m)², calculated based on information on adolescents' height and weight reported by their parents.

Table S6. Prevalence percentages/means, 95 % confidence intervals and p-values of test of proportion/t-test for selected characteristics of the study sample versus excluded cases, adolescents aged 11-17, KiGGS 2003-06

	Study sample (n=5251)	Excluded cases (n=1562)	Test of proportion/t-test
**Mean age (years)**	13.9 (13.8-13.9)	13.9 (13.8-14.0)	P=0.18
**Sex**			
boys (%)	51.0 (49.6-52.3)	52.2 (49.7-54-7)	P=0.41
**Region in Germany**			
former East Germany (%)	33.4 (32.2-34.7)	33.4 (31.1-35.8)	P=0.99
**Parental education (%)**			
primary	18.5 (17.5-19.6)	23.4 (21.1-25.6)	P<0.01
secondary	54.0 (52.6-55.3)	52.9 (50.3-55.6)	P=0.48
tertiary	27.5 (26.3-28.7)	23.7 (21.5-26.0)	P=0.01
number of missing cases	-	205	
**High physical-activity level (%)**	39.5 (38.2-40.8)	40.1 (37.3-42.9)	P=0.69
number of missing cases	-	391	
**High aerobic fitness (%)**	40.0 (38.7-41-4)	39.7 (35.9-43.4)	P=0.86
number of missing cases	-	904	
**High media use (%)**	36.7 (35.4-38.0)	43.6 (40.8-46.4)	P<0.01
number of missing cases	-	341	
**High energy expenditure (%)**	40.5 (39.2-41.8)	36.8 (33.7-40.0)	P=0.03
number of missing cases	-	626	
**High physical wellbeing (%)**	32.2 (30.9-33.4)	22.9 (20.8-24.9)	P<0.01
**Body Mass Index [a] (mean)**	20.9 (20.8-21.0)	21.1 (20.9-21.4)	P=0.02
number of missing cases	-	33	

[a] Body Mass Index = weight (kg) / height $(m)^2$, calculated based on information on adolescents' height and weight reported by their parents.
